# Multimodal Telerehabilitation in Post COVID-19 Condition Recovery: A Series of 12 Cases

**DOI:** 10.3390/reports8010035

**Published:** 2025-03-20

**Authors:** Beatriz Carpallo-Porcar, Esther del Corral Beamonte, Carolina Jiménez-Sánchez, Paula Córdova-Alegre, Natalia Brandín-de la Cruz, Sandra Calvo

**Affiliations:** 1 Department of Physical Therapy, Faculty of Health Sciences, Universidad San Jorge, Villanueva de Gállego, 50830 Zaragoza, Spain; 2 Aragón Health Research Institute (IIS Aragón), 50009 Zaragoza, Spain; 3 Hospital Royo Villanova, 50015 Zaragoza, Spain; 4 Department of Physiatry and Nursing, Faculty of Health Sciences, University of Zaragoza, 50009 Zaragoza, Spain

**Keywords:** post COVID-19 condition, long COVID, telerehabilitation, physiotherapy, rehabilitation, fatigue

## Abstract

**Background**: Post COVID-19 Condition is a recently recognized syndrome characterized by the persistence of various symptoms, including dyspnea, physical and mental fatigue, and post-exertional malaise. Currently, there is no established treatment or clear consensus on the effectiveness of rehabilitation, and given that patients could benefit from home-based rehabilitation, telerehabilitation, defined as remote rehabilitation using telematic systems, may be an option to reach more of the population with persistent COVID-19 symptoms. Therefore, it is necessary to show the efficacy of this telematic approach and the benefits of a multimodal rehabilitation strategy in these patients. **Methods**: Patients underwent home rehabilitation using a 12-week synchronous telerehabilitation system. The intervention included therapeutic education and physical and respiratory rehabilitation. The following variables were analyzed: Fatigue, quality of life, dyspnea, respiratory strength, aerobic capacity, and upper and lower limb strength. **Conclusions**: After 12 weeks, significant improvements were found in fatigue, aerobic capacity, and limb and respiratory strength. However, no improvement was found in dyspnea scores, which did not correlate with respiratory strength. Interestingly, a post-intervention correlation emerged between the distance covered in aerobic capacity and perceived fatigue, suggesting that asynchronous telerehabilitation could be a viable treatment strategy for these patients.

## 1. Introduction

The World Health Organization (WHO) defines Post COVID-19 Condition (PCC) as a condition characterized by “a history of probable or confirmed SARS-CoV-2 infection, usually 3 months from the onset of COVID-19, with symptoms that persist for at least 2 months and cannot be explained by an alternative diagnosis” [1,2]. PCC can be found in the literature as Long COVID, Long-haul COVID, or Post-Acute COVID syndrome. However, the term PCC is the one recommended in a Delphi consensus published in 2022 [1]. PCC persists beyond the acute phase, with symptoms recurring cyclically for months [1,3]. At least 10% of SARS-CoV-2 cases develop PCC [2]. Unlike patients who have developed sequelae, PCC usually affects individuals who present with mild to moderate acute symptoms but whose clinical condition progressively worsens [4]. PCC is a multisystemic syndrome with more than 150 symptoms described [1], which severely affects their functioning in everyday life. In fact, they have been reported to show a 60% decrease in their functional capacity [5]. Among the most impairment effects of PCC for patients are muscular fatigue and cognitive dysfunction [6,7]. In addition, it is associated with a wide range of symptoms, including dyspnea, “brain fog”, anxiety, depression, gastrointestinal disturbances, and overall reduced in quality of life [8,9]. Other key characteristic symptoms are exertion intolerance [10,11] and post-exertional malaise (PEM) [12]. These patients experience a deterioration in their daily activities and a socioeconomic impact, which in some cases leads to an inability to work.

There is currently no curative treatment. The WHO highlights the critical importance of rehabilitation for individuals with PCC, with physiotherapy playing a central role in patient recovery. The latest guidelines propose the need for a multimodal physiotherapeutic approach [13], combining dyspnea control, cardiorespiratory, and strength rehabilitation based on prior individual assessment with incremental exercise, even in cases of moderate PEM [14]. In addition, it is essential to implement therapeutic education strategies and adopt a comprehensive approach focused on improving lifestyle, nutrition, and self-management of the disease [15]. The current evidence recommends these multimodal approaches and even in telematic format [16]. However, the great diversity of symptoms mentioned and the irregularity in the intensity of them, require personalized interventions that cannot be fully covered by the care system. Telerehabilitation, therefore, proves to be particularly relevant for these patients [5]. Telerehabilitation makes it possible to reach people in different places and with different limitations to access rehabilitation centers and allows the patient to adapt their program to their available time [17]. Telerehabilitation systems have already proven to be effective for patients with various disabilities and even in patients with PCC [18]. Specifically, the asynchronous modality has also shown its effectiveness in post-COVID-19 patients, suggesting that it could also be a valid approach for individuals with PCC [18,19,20,21]. Although the existing literature on the multimodal approach in PCC patients treated with asynchronous telerehabilitation is limited, and the available studies report variable outcomes and low adherence rates, which makes it difficult to determine the real effect of these interventions, this modality remains a promising strategy worth further exploration. In addition, no studies have been found in the literature using asynchronous systems with program execution monitoring that would allow remote customization of the prescribed program. Therefore, if the efficacy of this system were to be shown, further studies could be carried out to open a window of personalized treatment adjusted to patients with PCC and other chronic diseases. For all these reasons, it is essential to propose the study whose hypothesis would be that a multimodal intervention of 12 weeks of therapeutic exercise and education through asynchronous telerehabilitation is beneficial in patients with PCC. Therefore, the primary aim of this study is to determine the preliminary efficacy of a multimodal approach using an asynchronous telerehabilitation system in improving fatigue, quality of life, and functional status variables, including aerobic capacity, strength, and maximal inspiratory and expiratory pressures in patients with PCC. The secondary aim is to explore potential correlations between functional variables (aerobic capacity and maximal pulmonary pressures) and self-reported outcomes (fatigue and dyspnea).

## 2. Material and Methods

### 2.1. Study Design and Population

This study is a prospective case series of 12 patients enrolled between February 2023 and April 2024 after each patient had signed the informed consent form on the first day of inclusion. Patients were recruited from three health centers in Aragón (Spain), and the intervention was approved by the Ethics Committee of Aragón (reference number: PI22/335).

### 2.2. Elegibility Criteria

The inclusion criteria were: (1) Aged 18 to 70 years; (2) Diagnostic criteria for having had COVID-19; (3) PCC symptoms for more than 12 weeks from the end of the acute phase; (4) Fatigue > 4 points on the Fatigue Severity Scale and (5) Independent locomotion. The exclusion criteria were: (1) Neurological disease preventing them from following the program; (2) Respiratory failure: SaO_2_ < 90% or respiratory rate ≥ 30; (3) Rheumatic diseases or acute musculoskeletal injuries that contraindicates exercise; (4) Not daily access to the Internet; (5) Unable to follow oral and written instructions in Spanish; (6) Patients who had participated in another study for the treatment of symptoms derived from COVID-19 in the last 4 weeks. Participants with an adherence rate of less than 90% to the intervention or who changed their medication during the intervention were considered to have dropped out. Adherence was recorded using the telerehabilitation application, which records the number of entries for each exercise. In addition, each patient was asked to report the time, sets, repetitions, and perceived fatigue each day, allowing daily monitoring of adherence to the program.

### 2.3. Procedure

After recruiting participants, the assessment team and the physiotherapist responsible for the intervention visited each health center to conduct both the initial and final evaluations. In the first phase, participants completed the written scales, followed by the physical examination. A physiotherapist then installed the application on their cell phone and explained how it worked.

Patients received biweekly calls to monitor their telerehabilitation treatment. Once the intensity of the proposed exercise decreased from 4 on the Borg scale, the program continued. If any discomfort or incidence occurred, the program was modified and individualized via the application. During the calls, participants were reminded to register their adherence to the program on the platform. In addition to these calls, an instant messaging channel was available to participants via the same application, allowing them to be in constant contact with the physiotherapist responsible for the intervention.

### 2.4. Intervention

The intervention consisted of a 12-week rehabilitation program carried out at home, 5 days a week, using asynchronous telerehabilitation via a free platform for patients (HEFORA). This app allows patients to receive a personalized rehabilitation prescription through explanatory videos and detailed descriptions. Physiotherapists can customize aerobic and strength exercises, adjusting sets, repetitions, speed, and specific days. Each video includes a voiceover to guide the patient through the exercises.

The multimodal program comprised three blocks of content based on the main current recommendations for rehabilitation treatment in PCC patients [14,15,16,21,22,23]. The program consisted of therapeutic education, pulmonary rehabilitation, and physical rehabilitation. Current evidence on the rehabilitation of patients with PCC recommends these multimodal approaches. In addition, cardiovascular and strength training seems to improve the physical and immunological variables of these patients [22], so this intervention was based on the current rehabilitation recommendations based on the initial and individualized assessment of each patient [14] (Supplementary Material S1).

#### 2.4.1. Therapeutic Education

This part consisted of three groups of videos. The first one was composed of several videos with general recommendations for PCC. Another group included explanatory videos for the proper organization and execution of the home program. The last group of videos featured exercises for self-management of symptoms. In the first group, patients had videos on day-to-day fatigue management, nutritional recommendations, rest and sleep recommendations, as well as relaxation techniques through breathing. In the second group, they had videos on self-management of the program with detailed explanations on the Borg scale, how to increase sets and repetitions, what to do in case of symptoms, or how to progress with the breathing exercises. Finally, for those cases with dyspnea, there were videos with explanatory exercises on how to manage it. Those with cough had a video on secretion clearance and cough management, and those with pain had videos on pain control and self-efficacy. The educational videos were available for viewing over the 12 weeks.

#### 2.4.2. Pulmonary Rehabilitation

The program included exercises designed to enhance lung capacity and respiratory strength. Each patient was prescribed three exercises to be performed once daily, with two sets of 10 repetitions per exercise, aiming for a perceived exertion of 2–3 on the modified Borg scale.

#### 2.4.3. Physical Rehabilitation

Based on the initial assessment and follow-up calls, each patient was individually prescribed specific materials from the available resources. The physical rehabilitation program consisted of aerobic and strength training divided into three intensity levels. Each level was divided into two phases: an initial adaptation phase followed by an improvement phase. Aerobic rehabilitation was performed 5 days per week. Patients were assigned to intensity levels based on their 6-min walk test (6 MWT) results. Those scoring ≤ 60% of their individual reference started at Level 1, those scoring between 61 and 70% started at Level 2, and those scoring > 70% started at Level 3. Progression through levels was determined by improvements in fatigue perception. The strengthening section focused on the major muscle groups: calves, hamstrings and glutes, quadriceps, abdominal and lumbar, shoulders, biceps and triceps, and pectorals, through 5 functional exercises. This block was performed three days per week with at least one rest day in between. Similar to the aerobic section, strength training was divided into three difficulty levels. All participants started at Level 1, performing 3 sets of 8 repetitions of each exercise, and gradually progressed to 3 sets of 15 repetitions, maintaining a fatigue level between 5 and 7 on the Borg scale.

### 2.5. Outcomes

#### 2.5.1. Primary Outcome

Fatigue was measured using the Fatigue Severity Scale (FSS) [24], a self-reported scale that assesses the severity of fatigue as a sense of physical tiredness, muscle weakness, and lack of energy. It has been validated in post-COVID-19 patients [25,26]. The scale is composed of 9 items with scores ranging from 1 = strongly disagree to 7 = strongly agree. The higher the number, the greater the severity of fatigue. The most common cut-off point is a mean score of 4 points, considering equal or more than 4 as severe fatigue [26].

#### 2.5.2. Secondary Outcomes

• Quality of life: EQ-5D-5L

The EuroQol 5-Dimension 5-Level Questionnaire (EQ-5D-5L) is a generic instrument for measuring health-related quality of life that has been validated in Spanish [27] and in PCC patients. It consists of two components: the levels of severity by dimensions and a visual analog scale (VAS) on general health. The descriptive system contains five dimensions of health (mobility, self-care, activities of daily living, pain/discomfort, and anxiety/depression), and each of them has five levels of severity (From 1 = No problem to 5 = Unable to perform). Participants indicate their perceived overall health on a VAS from 0 (“worst health I can imagine”) to 100 (“best health I can imagine”).

• Dyspnea

The Modified Medical Research Council (mMRC) [28] is a scale that has already been used in other studies to assess the degree of dyspnea in post-COVID-19 patients. This scale determines the magnitude of the dyspnea that the patient experiences, with scores ranging from 0 = absence of dyspnea to 4 = unable to leave the house due to dyspnea.

• Respiratory strength

Maximum Inspiratory Pressure (MIP) and Maximum Expiratory Pressure (MEP) can help evaluate respiratory muscle weakness in PCC patients [29] and provide the cut-off points to detect muscle weakness for the Spanish population [30,31]. MIP/MEP was measured based on the ATS/ERS 2002 guidelines using the MicroRPM CareFusion pressure meter with individual antibacterial filters [32,33,34].

• Functional status

- The Six Minutes Walk Test (6MWT) is a sub-maximal exercise test used and recommended to assess the maximum distance possible for 6 min in a 30-m corridor, allowing the patient to rest as needed. It has been shown to be reliable [35,36,37]. The distance covered by each participant is compared with the estimated distance for their gender, weight, and age according to the Troosters equation [38].
- The 30″ Sit to Stant Test (STST) is part of the Senior Fitness Test (SFT) designed by Rikli and Jones. It has been used as a stand-alone test, especially to assess weakness in respiratory patients who have passed COVID-19 [39,40,41]. For the 30″ STST, participants were seated in a chair with their feet flat on the floor and arms crossed over their shoulders, performing as many squats as possible within 30 s [42].
- The 30″ Arm Curl Test (ACT) is part of the SFT and is also used as a stand-alone test to assess strength. It has been shown to be reliable [39,40] in deconditioned patients and post-COVID-19 patients [43]. The higher the number of repetitions, the better the strength. During the 30″ ACT, participants were seated in a chair and performed as many elbow flexions as possible within 30 s using their dominant arm, with women lifting a 2 kg weight and men a 4 kg weight.

### 2.6. Statistical Analysis

Statistical analysis has been performed with IBM-SPSS Statistics software version 29. A significant level of 95% was assumed (*p* < 0.05). The variables have been described as mean and standard deviation (SD) or median and interquartile range. The Shapiro–Wilk test was used to determine the normality of the data. Intra-group categorical data (FSS, EQ-5D-5L, MIP/MEP, 6MWT, 30″ STST, and 30″ ACT) were measured with the T-Student test for related samples. Qualitative outcome (mMRC) was measured with McNemar’s Test. The effect size was calculated using Cohen’s d to determine clinical significance: negligible, small, medium, and large differences will be reflected in effect sizes of <0.2, 0.2–0.5, 0.6–0.8, and >0.8, respectively. A correlation analysis was conducted between dyspnea severity (measured by the mMRC scale) and MIP-MEP, as well as between the total distance covered in the 6MWT and fatigue severity (assessed by the FSS). Pearson’s correlation test was used to evaluate these relationships. In addition, the eta squared shall be calculated (η^2^) to quantify the proportion of the total variability in the measures. The eta squared values shall be interpreted according to the following criteria: 0.01 (small effect), 0.06 (medium effect), and 0.14 (high effect).

## 3. Results

Of the 12 cases included in this study, 9 were female and 3 were male, with an average age of 51 years. All of them had a medium-high educational level, were overweight according to Body Mass Index (BMI), and half of them had pathologies related to metabolic syndrome in their medical history. Two of them had emotional disorders, two were diagnosed with depression, and the other had anxiety. All of them have had symptoms of PCC for more than a year (Table 1 and Table 2). Within the symptomatology of PCC, all patients presented fatigue, and all but 2 participants had musculoskeletal pain with arthralgia and myalgia. After the musculoskeletal symptoms, neurological symptoms derived from neuroinflammation, mainly brain fog and memory loss, stand out in these patients. The third group of symptoms is respiratory symptoms, mainly dyspnea. All symptoms can be seen in Table 3. After 12 weeks of a multimodal intervention program with asynchronous telerehabilitation, the group showed a significant reduction in fatigue (FSS) score, decreasing from 6.41 to 4.85, indicating a very large effect size (d = 1.088). Regarding secondary variables, while no significant changes were observed in the self-perception test, clinical improvements were noted in both quality-of-life measures (EQ-5D-5L: coefficient and VAS) and in the degree of perceived dyspnea (mMRC). Functional capacity variables demonstrated statistically significant improvements across all measures after the intervention (6MWT: *p* < 0.001, d = 1.488; 30″ STST: *p* < 0.001, d = 1.127; 30″ ACT: *p* < 0.001, d = 1.422). Significant improvements were also found in the pulmonary pressure test (MIP: *p* = 0.003; d = 1.1.03; MEP: *p* = 0.031; d = 1.337). All measured variables exhibited very large clinical changes. (Table 4)

**Figure 1:**
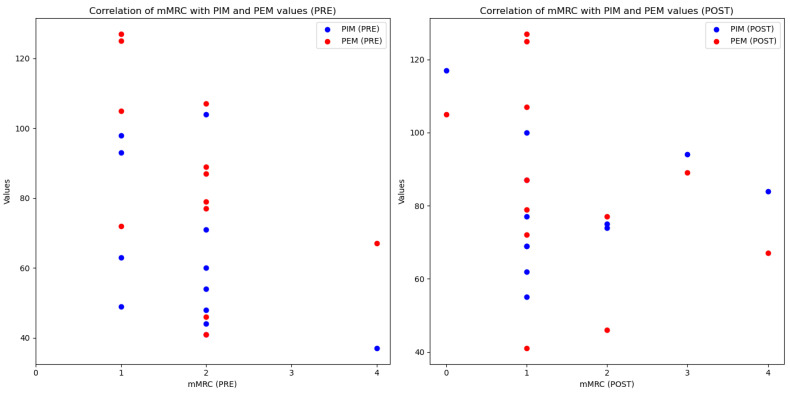
Correlation between dyspnea (mMRC) and PIM-PEM.

**Figure 2:**
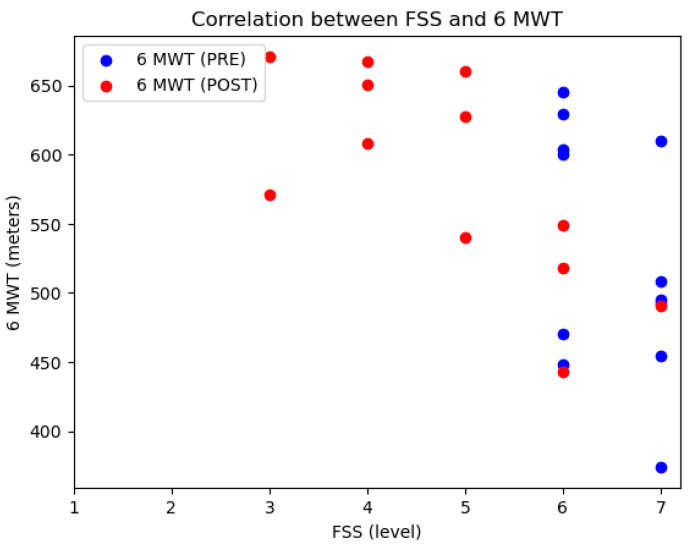
Correlation between fatigue (FSS) and meters (6 MWT).

Before the intervention, lower MIP values were observed without a correlation between MIP and mMRC (Figure 1). After the intervention, despite improvements in MIP and MEP values, the results by dyspnea groups did not show a correlation between the level of dyspnea and the respiratory strength values with a small and moderate effect (MIP, *p* = 0.887, η^2^ = 0.002; MEP, *p* = 0.131, η^2^ = 0.120) (Figure 1). Before the intervention, a shorter distance was covered by those with an FSS of 7 compared to those with a perceived fatigue level of 6. However, no correlation was found between these values but a moderate effect (*p* = 0.207, η^2^ = 0.215) (Figure 2). After the intervention, a correlation and a high effect was found between the distance walked in the 6MWT and perceived fatigue (*p* = 0.012; η^2^ = 0.505) (Figure 2).

## 4. Discussion

The study aimed to evaluate the effectiveness of a 12-week asynchronous multimodal telerehabilitation program for patients with PCC, focusing on improvements in fatigue, aerobic capacity, and limb and respiratory strength. After 12 weeks, there was a statistically significant improvement in the main variable, fatigue, and in the other physical status variables, although there were no improvements in quality of life and perception of dyspnea. Furthermore, although there was no correlation between the change in respiratory pressure and perceived dyspnea, at the end of the intervention, a correlation was found between the distance covered in the walking test and fatigue severity. These findings support our hypothesis that asynchronous telerehabilitation may be an effective treatment strategy for patients with PCC, particularly for enhancing physical function and reducing fatigue. The latter suggests that although multimodal physiotherapy may be effective in improving the functional capacity of these patients and decreasing their limitations, there may be disease-specific immunologic and inflammatory factors that perpetuate the symptoms and perceptions of the disease beyond functionality [44]. In fact, there are several studies that speak of the existence of a disproportionate and persistent inflammatory cascade that also occurs at the cerebral level. This neuroinflammation can contribute to symptoms such as the perception of fatigue, dyspnea, or malaise [45,46].

In terms of fatigue, there was a change in FSS, with a significant improvement in symptoms. The study by Groenveld et al. [47], which analyzed a single cohort group undergoing a 6-week virtual reality rehabilitation program, also found an improvement in fatigue. However, the Borg scale was used in this study. The difference between the two scales means that the result analyzed is not the same, as the Borg scale gives information on the fatigue perceived at a specific moment, while the FSS tells us how this fatigue affects the life of the patients. This makes our results more clinically relevant in terms of fatigue.

The most recent systematic review of telerehabilitation treatment for PCC patients [20] identified the use of online rehabilitation as an effective approach for improving physical symptoms and quality of life. Of the 6 clinical trials analyzed in the review, but with different methods and a diverse population, the population is not comparable. Despite these findings, no significant changes in quality of life were found in our study. Similar to the study by León-Herrera [48], which employed a mixed telerehabilitation approach (synchronous and asynchronous) aimed at improving quality of life and only reported improvements in the mental health category of the SF-36 scale after three months, compared to conventional treatment. The systematic review of Huang et al. [18] concluded that telerehabilitation showed no change in patients’ quality of life compared to other treatments. These mixed results suggest that the impact of telerehabilitation on quality of life may depend on factors such as intervention design, population characteristics, and measurement tools.

In terms of functional capacity, a significant improvement was found in the 6MWT. This represents not only a statistically significant change but also a clinically relevant improvement, surpassing the increase of the 30-m threshold for respiratory patients and even the increase of the 50-m benchmark established for non-respiratory conditions [49]. In the study of Groenveld et al. [47], in which they also analyzed a single group with telerehabilitation (but in virtual reality format) after 6 weeks, they also found significant changes in this variable. Their sample of 47 participants strengthens our findings. In addition, their program also included incremental physical exercise at three levels of difficulty. The big difference, in addition to the virtual reality format, was that their patients, although diagnosed with PCC, were recruited from 2020 at the onset of the disease and had an average duration of 7.2 months of evolution. In contrast, the participants in our study had been showing symptoms for more than a year, in very chronic stages of the disease, which could affect the outcome.

Significant improvements were also noted in the 30″ STST and 30″ ACT. In the study of León-Herrera et al. [48], which compared conventional treatment with the same intervention plus a multimodal telerehabilitation program, no significant changes were found in the squat test. This could be explained because their intervention was not specifically focused on improving physical fitness, but the content was more oriented towards self-management recommendations and synchronous group sessions. In contrast, in the study of Groenveld et al. [47], in which they performed specific exercises, significant improvements were found in the squat test, similar to our results. This suggests that asynchronous telerehabilitation can be an effective approach, provided that specific rehabilitation exercises are prescribed for each goal and each patient on an individual basis.

In terms of pulmonary rehabilitation variables, our study showed improvements in both inspiratory and expiratory strength with clinically meaningful changes. Wen et al. [50] studied discharged post-COVID-19 patients and initially found a relationship between MIP/MEP values and dyspnea levels measured with the mMRC scale. After an intervention using synchronous telerehabilitation via video chat, they found statistically significant changes in both the MIP/MEP variable and the mMRC. Similarly, the systematic review of Oliveira et al. [21] concluded that telerehabilitation improves variables such as fatigue but has limited impact on dyspnea, although this study only shows the changes compared to the face-to-face intervention and not the intra-group changes as in our case. In the study of Vallier et al. [51], in which two groups were also compared, one for physical rehabilitation in person versus the other via videoconference, no changes were found between groups, but statistically significant changes were found in the telerehabilitation group for dyspnea measured with the same scale. The difference, like other studies, is that it focuses on Post-Acute COVID patients, in whom better changes can be expected than in patients with chronic PCC. This difference in dyspnea results could be due to population differences, as patients in an acute or subacute stage respond better than chronic patients, such as those with chronic PCC. Additionally, it may also be influenced using the mMRC scale, which, as a subjective measure, may not fully capture subtle intra-group changes over time, particularly in a chronic population.

The main limitation is the impossibility of extrapolating the results outside our geographical area or in the general population with PPC. As this study is based on a brief review of 12 cases, so this small sample size limits the generalizability of the findings. Although the cases provide important insights, further research with a larger and more diverse sample is needed to confirm and extend these observations. Another limitation of this study is the lack of follow-up data to assess the long-term effects observed. To address this, studies with medium- and long-term follow-up periods are recommended. Our study paves the way for further research into the effectiveness of multimodal physiotherapy approaches delivered through asynchronous telerehabilitation in patients with chronic PCC. This approach shows promise in improving fatigue and functional capacity while offering the advantage of scalability, as it eliminates the need for live interventions, making it accessible to a larger and more diverse patient population.

## 5. Conclusions

A multimodal rehabilitation approach consisting of therapeutic education and physical and pulmonary rehabilitation, applied through asynchronous telerehabilitation, could be effective in reducing fatigue and improving functional and respiratory capacity in patients with PCC. This model offers a flexible and accessible rehabilitation option, particularly for individuals facing mobility or geographical barriers. However, further studies are needed to confirm these findings and explore the broader applicability of this approach in different patient populations.

## Figures and Tables

**Table 1:** Baseline sociodemographic and health data (pooled data).

		**n = 12**
**Sociodemographic**		
Sex	n (%)	
Male		3 (25.00)
Female		9 (75.00)
Age	m (SD)	51 (8.00)
BMI (kg/cm)	m (SD)	27.69 (4.86)
Level of education	n (%)	
Primary		0 (0.00)
Secondary		6 (50.00)
University		6 (50.00)
**Health Data**		
Time with symptoms (days)	n (%)	>365 (100.00)
Emotional disorder	n (%)	2 (16.70)
Other pathologies		
Diabetes		2 (16.70)
Obesity		1 (8.30)
Dyslipidemia		1 (8.30)
Hypertension		2 (16.70)
No pathology		6 (50.00)

BMI = Body Mass Index.

**Table 2:** Baseline sociodemographic and health data of each case.

Nº Case	Age	Gender	BMI	Level of Education	Metabolic Syndrome	Emotional Disorder
Case 1	35	Female	30.86	University	Obesity	Anxiety, Depression
Case 2	54	Female	30.03	Secondary	Obesity	None
Case 3	52	Female	32.88	Secondary	Obesity, Hypercholesterolemia	None
Case 4	52	Female	29.86	University	Hypertension	None
Case 5	39	Female	21.91	Secondary	None	None
Case 6	63	Male	31.12	Secondary	Hypertension, Obesity	Depression
Case 7	47	Female	20.7	University	None	None
Case 8	58	Female	23.53	University	Diabetes T2	None
Case 9	57	Male	35.27	Secondary	Obesity, Diabetes T2	None
Case 10	51	Female	23.73	University	None	None
Case 11	56	Female	29.62	University	Hypercholesterolemia	None
Case 12	47	Male	22.78	Secondary	None	None

BMI = Body Mass Index.

**Table 3:** Symptomatology.

Nº Case	General	Respiratory	Cardiovascular	Neurological	Gastrointestinal	Musculoskeletal	Oto-Laryngology
Case 1	Fatigue	None	None	None	None	Arthralgia, Myalgia	None
Case 2	Fatigue, Profuse sweating	Cough, Dyspnea	POTS, palpitations	Headaches, paresthesias, brain fog, lack of concentration	Nausea, Diarrhea, Pyrosis	Arthralgia, Myalgia	Dizziness
Case 3	Fatigue	Cough, Dyspnea	None	Headaches, paresthesias	None	Arthralgia, Myalgia	Dysphonia, Dizziness
Case 4	Fatigue, Fever	Cough, Dyspnea	POTS, palpitations	Headaches, paresthesias, brain fog, lack of concentration	None	Arthralgia, Myalgia	None
Case 5	Fatigue	None	Palpitations	Headaches, paresthesias, brain fog, lack of concentration	Abdominal Pain, Diarrhea	Arthralgia, Myalgia	None
Case 6	Fatigue	Dyspnea	POTS	Paresthesias, Anosmia, Brain fog, Lack of concentration	None	Arthralgia, Myalgia	None
Case 7	Fatigue	Dyspnea	None	Headache, Paresthesias, Brain fog	Diarrhea	Arthralgia, Myalgia	Dizziness
Case 8	Fatigue	Dyspnea	None	Paresthesia, Brain fog	None	Arthralgia, Myalgia	None
Case 9	Fatigue	Cough, Dyspnea	Palpitations	Brain fog, Lack of concentration	None	Arthralgia, Myalgia	Dizziness
Case 10	Fatigue	Cough, Dyspnea	None	Headaches, paresthesias, Anosmia, Brain fog, Lack of concentration	Nausea, Diarrhea	Arthralgia, Myalgia	None
Case 11	Fatigue	Dyspnea	None	Anosmia, Brain fog, Lack of concentration	None	Arthralgia, Myalgia	None
Case 12	Fatigue	None	None	None	None	None	None

POTS = Postural orthostatic tachycardia.

**Table 4:** Changes after the intervention.

		**Pre-Intervention n = 12**	**Post-Intervention n = 12**	**Difference**	***p* Value**	**Effect Size Cohen’s d**
**Primary variable**						
FSS	m (SD)	6.41 (0.51)	4.85 (1.39)	−1.57 (1.44)	**0.003 ^T^**	1.088
**Secondary variables**						
EQ−5D						
Coefficient	m (SD)	0.57 (0.30)	0.74 (0,16)	0.17	0.246 ^W^	
VAS	m (SD)	56.08 (13.33)	60.00 (14.45)	−3.91 (12.33)	0.295 ^T^	0.317
mMRC	m (RIQ)	2 (1)	1 (1)	1.00	0.125 ^M^	
6 MWT	m (SD)	527.45 (87.08)	583.04 (75.99)	55.58 (37.34)	**<0.001 ^T^**	1.488
30″ STST	m (SD)	14.00 (4.00)	18.00 (4.00)	4.00 (4.00)	**<0.001 ^T^**	1.127
30″ ACT	m (SD)	16.00 (4.00)	21.00 (4.00)	5.00 (4.00)	**0.001 ^T^**	1.422
MIP	m (SD)	63.50 (21.16)	80.25 (17.29)	16.75 (15.20)	**0.003 ^T^**	1.103
MEP	m (SD)	85.17 (27.50)	99.33 (19.77)	14.17 (19.89)	**0.031 ^T^**	1.337

EQ-5D-5L = quality-of-life scale; mMRC = Medical Research Council dyspnea scale; 6 MWT = 6-min walk test; STST = sit-to-stand test; ACT = elbow flexion test; MIP = maximal inspiratory pressure; MEP = maximal expiratory pressure. W = Willcoxon test; T = Student’s tee; M = McNemar test. Bold=statistical significance.

## Data Availability

The data supporting the findings of this study have been provided for review. Due to privacy and protection concerns, the data cannot be made publicly accessible. This measure ensures the confidentiality and security of sensitive information. For this reason, the raw data will be available upon request from the corresponding author.

## Supplementary Materials

The following supporting information can be downloaded at: https://www.mdpi.com/article/10.3390/reports8010035/s1, Figure S1: Example of the program’s prescription interface; Figure S2: Example of a therapeutic education video; Figure S3: Example of a video of physical exercise.
