# Supplementary material for: Multimodal Telerehabilitation in Post COVID-19 Condition Recovery: A Series of 12 Cases
Source: Reports (MDPI). 2025 Mar 20;8(1):35. doi: 10.3390/reports8010035 (PMC12199987; doi:10.3390/reports8010035)

Supplement S1: examples of how the programme is prescribed.

Figure S1. Example of the program's prescription interface.

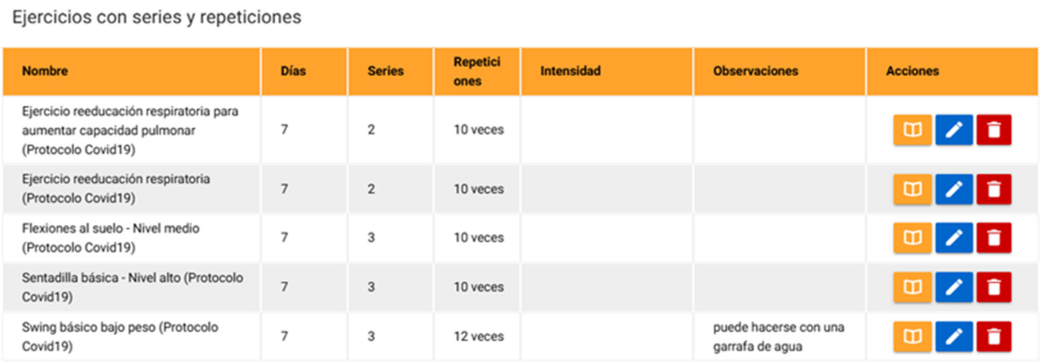
Ejercicios con series y repeticiones

Nombre	Días	Series	Repeticiones	Intensidad	Observaciones	Acciones
Ejercicio reeducación respiratoria para aumentar capacidad pulmonar (Protocolo Covid19)	7	2	10 veces			
Ejercicio reeducación respiratoria (Protocolo Covid19)	7	2	10 veces			
Flexiones al suelo - Nivel medio (Protocolo Covid19)	7	3	10 veces			
Sentadilla básica - Nivel alto (Protocolo Covid19)	7	3	10 veces			
Swing básico bajo peso (Protocolo Covid19)	7	3	12 veces		puede hacerse con una garrafa de agua	

Figure S2. Example of a therapeutic education video.

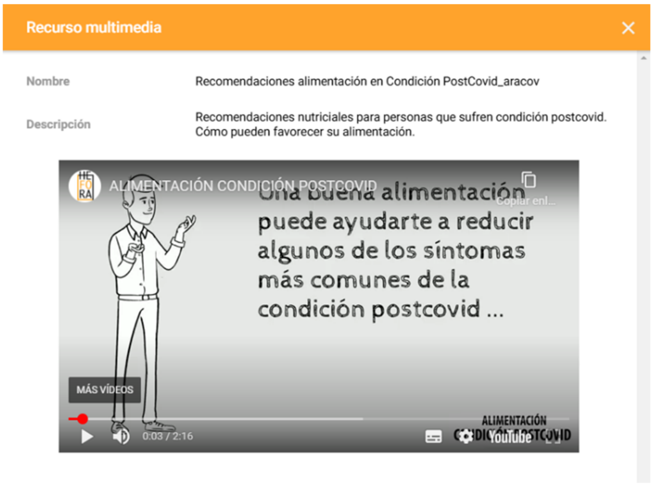

Figure S3. Example of a video of physical exercise.

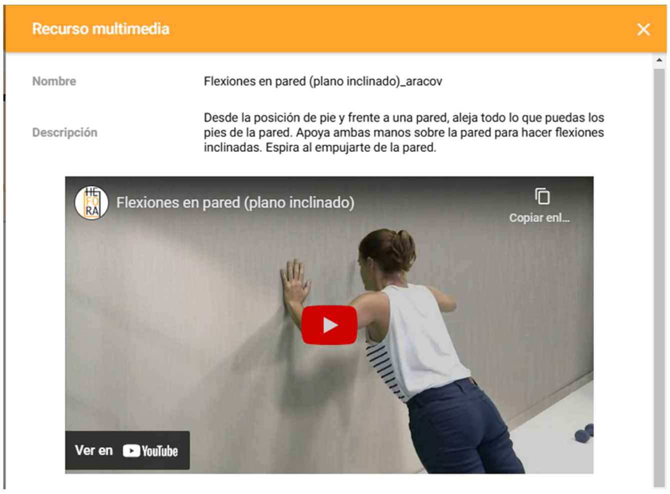

Supplement: Supplementary file 1 [file reports-08-00035-s001.zip › reports-3527273-supplementary.pdf]
